# Validation of the Japanese version of the CDC HRQOL‐4 in workers

**DOI:** 10.1002/1348-9585.12152

**Published:** 2020-07-27

**Authors:** Odgerel Chimed‐Ochir, Yuko Mine, Makoto Okawara, Koki Ibayashi, Fuyu Miyake, Yoshihisa Fujino

**Affiliations:** ^1^ Department of Environmental Epidemiology Institute of Industrial Ecological Sciences University of Occupational and Environmental Health Kitakyushu Japan; ^2^ Department of Preventive Medicine and Community Health School of Medicine University of Occupational and Environmental Health Kitakyushu Japan

**Keywords:** health‐related quality of life, reliability, unhealthy days, validity, workers’ health

## Abstract

**Background:**

We set out to investigate the reliability and validity of the Japanese version of the CDC core healthy day measures assessing health‐related quality of life (CDC HRQOL‐4) in Japanese workers.

**Methods:**

This cross‐sectional study was conducted among 1360 Japanese workers of one Japanese company located in Kyushu. Cronbach's alpha was calculated to evaluate the internal consistency of CDC HRQOL‐4 items. The concurrent validity was tested by assessing whether the CDC HRQOL‐4 items correlated well with the corresponding domains of the SF‐8 and the overall WFun score. The construct validity was tested by assessing the ability of the CDC HRQOL‐4 to discriminate groups with and without any current disease, pain, mental problem, and sleeping disorder.

**Results:**

Cronbach's alpha for three of the four CDC HRQOL‐4 items was 0.80, which is greater than the minimal standard (0.70) recommended for internal consistency reliability. Correlation coefficients ranging from 0.25 to 0.67 were obtained between CDC HRQOL‐4 items and the SF‐8 domains and WFun score. Workers with any current disease, mental problem, pain or sleeping disorder reported higher numbers of unhealthy days, and a higher odd of poor health than those without such problems. Japanese version of the CDC HRQOL‐4 shows a good concurrent validity with the SF‐8 and the WFun tool and good construct validity among Japanese workers in the current study.

**Conclusions:**

Japanese version of the CDC HRQOL‐4 is a reliable and valid instrument that may be used to assess overall health and physically and mentally unhealthy days in Japanese workers.

## 1 INTRODUCTION

The traditional measures of health, such as mortality and morbidity, reveal very little about other aspects that are considered to be important health domains, including physical functions, cognitive functions, and perceptions about health.^1^ Therefore, those who measure health outcomes have begun moving toward assessing a population's health not only on the basis of saving lives, but also in terms of improving the health‐related quality of life (HRQOL). HRQOL is intended to describe the interaction between the experiences associated with health problems and a patient's/population's perception of their health.^2^ Many researchers have applied various HRQOL instruments to measure the self‐assessed health status; notably, this status has been shown to be a more powerful predictor of mortality and morbidity than many objective measures of health, because people generally seek heath care when they feel unhealthy.^3^

In a previous study, we used the Japanese version of the CDC (Center for Disease Control and Prevention, USA) HRQOL‐4 tool for the first time in Japan.^4^ We applied this tool to study the relationship between HRQOL (poor perceived health/unhealthy days) and workers’ pain, but did not assess the validity of the tool at that time due to the wide and international acceptance of its construct and criterion validity.^5^, ^6^ We herein set out to investigate the reliability and validity of the Japanese version of the CDC HRQOL‐4 in Japanese workers to promote the application of tool among Japanese workers.

## 2 METHODS

### 2.1 Subjects

We distributed questionnaires to 2294 Japanese workers in Kyushu. Out of 2294 workers, 1360 workers responded to the questionnaire. The dataset is exactly same as we used in our previous work.^4^ The study was designed as cross‐sectional study. Participants were asked to fill out a self‐administered questionnaire. All participants gave their written informed consent for participating in the study. Data were collected during November and December, 2018. The study was approved by the ethical committee of the University of Occupational and Environmental Health, Japan.

### 2.2 HRQOL assessment tools

The CDC HRQOL‐4 was developed by the CDC of the USA. It fetches a self‐rated health status by asking about the following four domains^7^: (a) self‐rated health, (b) the number of physically unhealthy days in the past 30 days, (c) the number of mentally unhealthy days in the past 30 days, and (d) the number of days with activity limitation in the past 30 days. Self‐rated health is recorded as excellent, very good, good, fair, and poor.^7^ We further classified this into two levels, good (excellent, very good, good) and poor (fair, poor), for ease of statistical analysis. This is in line with similar studies in the literature^8^ and the recommendation of the Institute of Medicine (USA) that a population's self‐reported health should be assessed as a percentage of adults reporting fair or poor health.^9^ Total unhealthy days were computed by summing the number of days that each respondent reported being physically and/or mentally unhealthy, for a maximum of 30 days per person.^7^ The CDC HRQOL‐4 instrument is free for public use, not copyrighted and does not require permission for use or licensing fees. The CDC HRQOL‐4 tool was translated from English into Japanese by Japanese epidemiologist and occupational physician. Forward translations were reconciliated. After that, the back translation was performed by a native English‐speaking researcher to ensure the equivalence between the original English version and the Japanese translated version. No conceptual and contextual differences from original English version were found.

The Japanese version of SF (short form)‐8 Health Survey (SF‐8 Form)^10^ and the WFun tool^11^ were used as confirmatory tools to assess construct validity of CDC HRQOL‐4. We obtained the permission from Certified NPO Institute for Health and Medical Evaluation iHOPE International to use SF‐8 Form. It is comprised of eight questions on quality of life.

The WFun tool, which is a work functioning impairment scale, was originally developed in Japanese by Fujino et al to measure the ability to function at work and reflects the social, physical and mental problems of workers. It consists of seven self‐assessment questions, each of which is scored from 1 (best health) to 5 (worst health).^11^ Thus, the total WFun score ranges from 7 (best health) to 35 (worst health). The total summed raw score of the seven attributes can be considered a “sufficient statistic,” since Rasch model^12^ was used to develop the WFun tool. This tool has proven useful in detecting health problems that affect working ability.^13^

### 2.3 Other variables

Pain was evaluated as described in our previous work.^4^ For the present study, workers’ pain was divided into two groups: mild (mild and moderate) and severe. Participants were asked if they were being treated for any current disease, including a depression and other mental problems. Sleeping disorder was assessed by Japanese version of Athens insomnia scale which was developed and validated in 2013.^14^ Respondents scored each item from 0 (the best) to 3 (the worst), and sleep was considered to be normal (no sleeping disorder) if the total score was less than 4. Those with scores over 5.5 were considered to have a sleeping disorder.^14^

### 2.4 Data analysis

The demographic characteristics of respondents are described with numbers and percentages. The percentages of missing answers on HRQOL‐4 items were assessed to evaluate the feasibility of the CDC HRQOL‐4 instrument. Cronbach's alpha was calculated to evaluate the internal consistency of CDC HRQOL‐4 items. The concurrent validity was tested by assessing whether the CDC HRQOL‐4 items correlated well with the corresponding domains of the SF‐8 and the overall WFun score. Spearman's rank correlation coefficient was used. The construct validity was tested by assessing the ability of the CDC HRQOL‐4 to discriminate groups with and without any current disease, pain, mental problem, and sleeping disorder. The logistic and linear regression models were used to check construct validity and adjusted by age, sex, and work type. Significance was set at the 5% level (*P* < .05). SAS Version 9.4 (SAS Institute, Inc) was used to analyze the data.

## 3 RESULTS

### 3.1 Response

We distributed questionnaires to 2294 participants and 1597 (70%) of them responded. After excluding cases with missing values for CDC‐HRQOL‐4 variables, SF‐8 variables, and WFun variables, a total of 1360 Japanese workers are analyzed in this study.

The mean age of the participants was 43.7 ± 12.8; 58% were men and 42% were women. White‐, pink‐, and blue‐collar workers represented 54%, 26%, and 20% of all participants, respectively.

All of the CDC HRQOL‐4 items had less than 3% missing answers, with the missing answers by item ranging from 1% for self‐rated health to 3% for mentally unhealthy days and days with activity limitation.

### 3.2 Internal consistency

Cronbach's alpha of the three items of CDC HRQOL‐4 (physically and mentally unhealthy days, activity limitation days) was 0.80. The item of “self‐rated health” was omitted from analysis because of its dichotomized scaling.

### 3.3 Concurrent validity

The correlations between the CDC HRQOL‐4 and the SF‐8 domains and WFun score are shown in Table 1. Correlation coefficients ranging from 0.25 to 0.67 were obtained between CDC HRQOL‐4 items and the SF‐8 domains and WFun score, and no negligible correlation was observed. The self‐rated health item of the CDC HRQOL‐4 showed the highest correlation with the “general health” (*ρ* = 0.67, 95% CI 0.64‐0.70, *P* < .001) and “vitality” (*ρ* = 0.68, 95% CI 0.65‐0.71, *P* < .001) domains of the SF‐8. In general, the items of the CDC HRQOL‐4 showed the highest correlations with the corresponding domains of the SF‐8. For example, the physically unhealthy days item of the CDC HRQOL‐4 had the highest correlation with general poor health and the “physical function” domain on the SF‐8. Similarly, the mentally unhealthy days item on the CDC HRQOL‐4 was highly correlated with “mental health” and “role emotional” domain of the SF‐8. For the WFun tool, poor self‐rated health (*ρ* = −0.45, 95% CI −0.49 to −0.41, *P* < .001) total unhealthy days (*ρ* = −0.43, 95% CI −0.47 to −0.38, *P* < .001), and activity limitation (*ρ* = −0.37, 95% CI −0.42 to −0.33, *P* < .001) on the CDC HRQOL‐4 were negatively correlated with good work functioning on the WFun tool.

**TABLE 1:** Concurrent validity of CDC HRQOL‐4 (Spearmen correlations between CDC HRQOL‐4 and SF‐8 & CDC HRQOL‐4 and Wfun)

CDC HRQOL‐4		Self‐rated health		Unhealthy days				Activity limitation days		Total unhealthy days	
				Physical		Mental					
SF Short Survey Form ‐8		*ρ*	95%CI	*ρ*	95%CI	*ρ*	95%CI	*ρ*	95%CI	*ρ*	95%CI
SF1	General health	.67	0.64‐0.70	.50	0.46‐0.54	.41	0.36‐0.45	.43	0.38‐0.47	.55	(0.51‐0.58)
SF2	Physical function	.40	0.35‐0.44	.43	0.39‐0.48	.21	0.15‐0.25	.31	0.26‐0.36	.35	(0.30‐0.40)
SF3	Role physical	.42	0.38‐0.47	.35	0.30‐0.40	.26	0.21‐0.31	.42	0.37‐0.46	.39	(0.34‐0.43)
SF4	Bodily pain	.44	0.40‐0.49	.40	0.35‐0.44	.26	0.22‐0.32	.32	0.29‐0.39	.43	(0.39‐0.48)
SF5	Vitality	.68	0.65‐0.71	.43	0.38‐0.47	.41	0.36‐0.45	.39	0.34‐0.43	.51	(0.47‐0.55)
SF6	Social functioning	.47	0.42‐0.51	.35	0.30‐0.39	.34	0.29‐0.39	.41	0.35‐0.45	.42	(0.38‐0.47)
SF7	Role emotional	.57	0.54‐0.61	.25	0.20‐0.30	.58	0.54‐0.61	.36	0.33‐0.42	.50	(0.46‐0.54)
SF8	Mental health	.52	0.48‐0.56	.30	0.25‐0.35	.47	0.43‐0.51	.43	0.40‐0.48	.47	(0.43‐0.51)
Wfun	Overall WFun score	−.45	(−0.49‐0.41)	−.27	(−0.33‐0.23)	−.45	(−0.48‐0.39)	−.37	(−0.42‐0.33)	−.43	(−0.47‐0.38)

Note

All correlations were at significant level of <0.0001.

### 3.4 Construct validity

Table 2 shows the ability of the CDC HRQOL‐4 items to discriminate between respondents with and without health problems. In general, workers with any current disease, mental problem, pain, or sleeping disorder reported higher numbers of physically unhealthy days, mentally unhealthy days, and activity limitation days than those without such problems after adjusting with age, sex, and work type. All of these differences were statistically significant (*P* < .0001). For example, workers with depression or other mental problems reported 13.42 more unhealthy days than those without a mental problem (*P* < .0001).

**TABLE 2:** Construct validity of the CDC HRQOL‐4 (Relationship between HRQOL and some health conditions)

CDC HRQOL‐4		Self‐rated health^a^				Unhealthy days								Total unhealthy days^b^				Activity limitation days^b^			
		Poor health (%)	AOR	95% CI	*P*‐value	Physical^b^				Mental^b^											
Groups						Number of workers	PE	95% CI	*P*‐value	Number of workers	PE	95% CI	*P*‐value	Number of workers	PE	95% CI	*P*‐value	Number of workers	PE	95% CI	*P*‐value
Current disease	No	26.5	Reference			932	Reference			930	Reference			929	Reference			932	Reference		
	Yes	49.2	2.15	(1.66‐2.79)	<.0001	334	3.75	(2.93‐4.58)	<.0001	327	2.31	(1.51‐3.11)	<.0001	326	4.69	(3.62‐5.76)	<.0001	326	2.56	(1.89‐3.27)	<.0001
Depression or other mental problem	No	40.9	Reference			306	Reference			305	Reference			304	Reference			305	Reference		
	Yes	77.8	5.13	(2.15‐12.22)	.0002	28	6.06	(3.66‐8.45)	<.0001	28	12.87	(1.69‐15.05)	<.0001	27	13.42	(10.43‐16.42)	<.0001	28	8.07	(6.13‐10.00)	<.0001
Presence of pain	No	14.7	Reference			432	Reference			432	Reference			432	Reference			432	Reference		
	Yes	41.5	3.14	(2.24‐4.39)	<.0001	900	2.4	(1.50‐3.31)	<.0001	890	1.78	(1.0‐2.65)	<.0001	888	3.72	(2.56‐4.88)	<.0001	891	1.36	(0.62‐2.11)	.0003
Pain intensity	Mild	36.6	Reference			696	Reference			690	Reference			690	Reference			689	Reference		
	Strong	52.9	2.21	(1.64‐3.00)	<.0001	201	3.4	(2.42‐4.37)	<.0001	198	2.53	(1.59‐3.46)	<.0001	196	4.83	(3.59‐6.09)	<.0001	199	2.09	(1.29‐2.90)	<.0001
Sleeping disorder	No	28.9	Reference			1240	Reference			1231	Reference			1229	Reference			1232	Reference		
	Yes	86.6	13.9	(7.63‐25.29)	<.0001	91	6.6	(5.24‐7.97)	<.0001	90	7.86	(6.59‐9.14)	<.0001	90	11.24	(9.52‐12.96)	<.0001	90	6.83	(5.72‐7.93)	<.0001

Abbreviations: AOR, adjusted odds ratio (adjusted with age, sex, type of work); PE, parameter estimates (adjusted with age, sex, type of work).

^a^ Logistic regression analysis was conducted.

^b^ Linear regession analysis was conducted.

In the adjusted model, participants who reported health problems had significantly greater odds of having poor health compared to those without health problems. In particular, workers with sleeping disorder had significantly greater odds of having poor health (AOR = 13.89, 95% CI = 7.63‐25.29, *P* < .0001) than workers without sleeping disorder.

## 4 DISCUSSION

In this study, we examined the feasibility, validity, and reliability of the CDC HRQOL‐4 instrument among Japanese workers. The current study showed a high feasibility, with 1%‐3% of missing values for the HRQOL‐4 items. Previous studies also showed low percentages of missing values for all items of the HRQOL‐4 instrument.^6^, ^8^ Thus, our findings suggest that participants had no difficulty in answering the four items of the Japanese version of the HRQOL‐4.

In terms of reliability, the Cronbach's alpha for three of the four CDC HRQOL‐4 items was 0.80, which is greater than the minimal standard (0.70) recommended for internal consistency reliability.^15^ Several different versions of the HRQOL‐4 instrument have also yielded Cronbach's alpha values between 0.69 and 0.77 for internal consistency reliability.^6^, ^8^ Based on our findings, we believe that the Japanese version of the CDC HRQOL‐4 has acceptable internal consistency reliability among workers.

The current study found that the CDC HRQOL‐4 items showed weak (r = .25) to moderate correlation (r = .67) with domains of the SF‐8 and the WFun total score. In particular, the items of the CDC HRQOL‐4 showed the highest correlation with the corresponding domains of SF‐8, as mentioned in the Results section. Significant negative correlations were evident between good work functioning and poor self‐rated health and number of unhealthy days. There is no officially reported threshold of an acceptable correlation coefficient to guarantee the concurrent validity of the HRQOL‐4; however, previous studies considered correlation coefficients of 0.3‐0.7 to satisfactorily indicate concurrent validity.^5^, ^6^, ^8^ A previous report suggested that wide confidence interval do not allow a definitive conclusion about the strength of the relationship between the variables. For our study, the range of plausible values (95% CI) for the correlation coefficients was very narrow (Table 1), which supports the strength of the relationship between our study variables.^16^ Thus, we conclude that the HRQOL‐4 shows a good concurrent validity with the SF‐8 and the WFun tool in the current study.

Comparison of workers with or without current disease, mental problem, pain, or sleeping disorder produced evidence that the construct validity of the HRQOL‐4 tool is excellent. The highest differences were found between workers with and without a mental problem or sleeping disorder. Several studies also found that those with a mental problem had much greater odds of poor health, mentally unhealthy days, physically unhealthy days, and activity limitation^17^, ^18^ found that insufficient sleep was greatly associated with workers’ poor performance and poor health. Our group previously reported that poor self‐rated health and the number of unhealthy days were significantly associated with the presence and intensity of pain among Japanese workers.^4^ Several other studies^19^, ^20^ showed that persons with any disease/chronic condition reported significantly more physically unhealthy days, mentally unhealthy days, and activity limitation than those without any disease. Based on our present findings, we conclude that the Japanese version of the CDC HRQOL‐4 showed good construct validity among Japanese workers.

This study has several strengths and weaknesses that warrant mention. Regarding strengths, we note that the sample size of 1360 employees is relatively large for a validity study, and it is 2‐10 times higher than those used in other studies.^5^, ^6^, ^8^ In addition, this instrument takes about 1 minute to administer and may be completed via telephone. The scoring is very direct, as it is expressed by the absolute number of unhealthy days. This contrasts with most of the other relevant tools, which use summary scores and/or subscale scores based on psychometrically derived or preference‐based weighting^7^ and thus are less straightforward to interpret. In general, the analysis of this tool does not require any sophisticated statistical skill, thus, would be user friendly for occupational physicians. Regarding weaknesses, we did not examine test‐retest reliability, thus, may not fully assure that the measurement obtained is representative and stable over time. Second, we involved participants from only Kyushu region of Japan, which may have under‐represented the overall Japanese workers. Third, only 1.25% of those who were investigated in the current study were over 65 years old, thus, careful consideration is needed to apply this tool among older workers.

In sum, we herein conclude that the Japanese version of the CDC HRQOL‐4 is a reliable and valid instrument that may be used to assess overall health and physically and mentally unhealthy days in Japanese workers. Therefore, the Japanese version of the CDC HRQOL‐4 can be further used to track workers' health.

## DISCLOSURE

*Approval of the research protocol*: The study was approved by the ethical committee of the University of Occupational and Environmental Health, Japan on 5 April 2018. *Informed consent*: All participants gave their informed consent for participating in the study. *Registry and the registration no. of the study/trial*: N/A. *Animal studies*: N/A.

## CONFLICT OF INTEREST

Authors declare that they have no conflict of interest.

## AUTHOR CONTRIBUTIONS

OC analyzed the data and wrote manuscript; YM took responsibility of data cleaning and data entering; MO, KI, and FM reviewed manuscript; YF collected data and reviewed analysis and manuscript.
